# Metabolite Profiling of 14 Wuyi Rock Tea Cultivars Using UPLC-QTOF MS and UPLC-QqQ MS Combined with Chemometrics

**DOI:** 10.3390/molecules23020104

**Published:** 2018-01-24

**Authors:** Si Chen, Meihong Li, Gongyu Zheng, Tingting Wang, Jun Lin, Shanshan Wang, Xiaxia Wang, Qianlin Chao, Shixian Cao, Zhenbiao Yang, Xiaomin Yu

**Affiliations:** 1 College of Horticulture, Fujian Agriculture and Forestry University, Fuzhou 350002, China; cstc1990@hotmail.com (S.C.); limei123home@gmail.com (M.L.); zgy5403@126.com (G.Z.); lalaxiaojie0527@163.com (T.W.); yang@ucr.edu (Z.Y.); 2 FAFU-UCR Joint Center for Horticultural Biology and Metabolomics, Fujian Provincial Key Laboratory of Haixia Applied Plant Systems Biology, Fujian Agriculture and Forestry University, Fuzhou 350002, China; realnadal@163.com (J.L.); shanshanwang22@yeah.net (S.W.); wangxiaxia530@126.com (X.W.); 3 Wuyi Star Tea Industry Co., Ltd., Wuyishan 354300, China; chaoqianlin@wuyistar-tea.com (Q.C.); caoshixian@wuyistar-tea.com (S.C.); 4 Center for Plant Cell Biology, Institute of integrated Genome Biology, and Department of Botany and Plant Sciences, University of California, Riverside, CA 92521, USA

**Keywords:** Wuyi Rock tea, quality, UPLC-QTOF MS, UPLC-QqQ MS, metabolite profiling, metabolomics, cluster analysis, cultivars

## Abstract

Wuyi Rock tea, well-recognized for rich flavor and long-lasting fragrance, is a premium subcategory of oolong tea mainly produced in Wuyi Mountain and nearby regions of China. The quality of tea is mainly determined by the chemical constituents in the tea leaves. However, this remains underexplored for Wuyi Rock tea cultivars. In this study, we investigated the leaf metabolite profiles of 14 major Wuyi Rock tea cultivars grown in the same producing region using UPLC-QTOF MS and UPLC-QqQ MS with data processing via principal component analysis and cluster analysis. Relative quantitation of 49 major metabolites including flavan-3-ols, proanthocyanidins, flavonol glycosides, flavone glycosides, flavonone glycosides, phenolic acid derivatives, hydrolysable tannins, alkaloids and amino acids revealed clear variations between tea cultivars. In particular, catechins, kaempferol and quercetin derivatives were key metabolites responsible for cultivar discrimination. Information on the varietal differences in the levels of bioactive/functional metabolites, such as methylated catechins, flavonol glycosides and theanine, offers valuable insights to further explore the nutritional values and sensory qualities of Wuyi Rock tea. It also provides potential markers for tea plant fingerprinting and cultivar identification.

## 1. Introduction

Oolong tea is a partially-fermented tea manufactured in southeast China, mainly in Fujian and Guangdong. In recent years, the production and consumption of oolong tea has increased greatly worldwide, attributed to its pleasurable aroma and taste favored by consumers [1,2]. As a functional drink, oolong tea exhibits many health-promoting benefits, such as anti-oxidant, anti-cancer, anti-obesity, anti-atherosclerosis, anti-diabetes and anti-allergic activities [1].

Wuyi Rock tea is a distinctive and premium subcategory of oolong tea grown in Wuyi Mountain, which is a UNESCO World Heritage site and considered the birthplace of oolong tea, as well as nearby regions in the north part of Fujian Province. Recognized as the most prestigious oolong tea in China, Wuyi Rock tea boasts a history of over 1500 years and is renowned for its rich flavor and long-lasting fragrance, so-called ‘rock charm and floral fragrance’ [3]. Consumer demand for Wuyi Rock tea, both domestic and abroad, is increasing year by year but is often hindered by limited supplies and resulting high market price. Production of high-quality Wuyi Rock tea involves very complicated procedures, including leaf-picking, withering, zuoqing (partial fermentation, which includes alternating rotation and cooling steps), fixation (enzyme inactivation), rolling, roasting, grading and packaging [1] and usually relies on experienced workers [4]. Apart from manufacturing procedures, like the production of other types of tea, the quality of Wuyi Rock tea is also determined by the initial metabolite contents in fresh tea leaves, which depends on both cultivars and environmental factors [5,6,7]. According to the conventional classification by local people on the basis of the natural environment where tea plants have been grown, Wuyi Rock tea is subdivided into authentic rock tea, half rock tea, riverbank tea and tea grown outside the main production area in descending grade order [3]. This may suggest the geographic location as a key factor influencing the quality of Wuyi Rock tea. 

On the other hand, the choice of cultivars to produce Wuyi Rock tea also matters but remains underexplored except for a few comparative studies, which have focused only on a small number of major constituents in processed tea and were not performed under controlled environmental conditions [8,9]. Contributed by unique climate and soil conditions, Wuyi Mountain is home to a large collection of tea germplasms. Historically, some tea cultivars have been used to produce Wuyi Rock tea since ancient times. Primary cultivars are ‘Shuixian’ and ‘Rougui’; the former was registered as a national tea cultivar whereas the latter as a provincial tea cultivar due to their stable quality and higher yields. Other elite clonal cultivars include ‘Dahongpao’, ‘Tieluohan’, ‘Baijiguan’, ‘Shuijingui’, ‘Guazijin’ and ‘Jinsuoshi’, which are among the estimated 216 cultivars listed as Wuyi Rock tea cultivars. Such diverse genetic resources are valuable for producing Wuyi Rock tea. However, for most of these cultivars, research to examine quality-related traits at the genetic and metabolomic level is critical yet insufficient. Therefore, it would be helpful to comprehensively survey the metabolomes of representative tea cultivars, and identify important varietal differences relevant to tea quality. 

Non-targeted metabolomics approach based on UPLC-QTOF MS, GC-TOF MS or NMR is a powerful technique capable of detecting a high number of endogenous metabolites simultaneously [10]. It has been widely applied in tea research to study impacts of environmental factors on tea metabolites [11,12,13], characterize dynamic changes during tea manufacture [14,15] and discover key compounds for tea type discrimination [16,17]. In this study, by combining UPLC-QTOF MS-based non-targeted analysis with UPLC-QqQ MS-based targeted quantifications of catechins, rutin, amino acids and caffeine, we analyzed the metabolite profiles of unprocessed fresh tea leaves of 14 major Wuyi Rock tea cultivars grown in the same environmental conditions subjected to the same cultivation practices. Data processing by principal component analysis (PCA), partial least squared discriminant analysis (PLS-DA) and hierarchical cluster analysis revealed differences as well as commonalities between the leaf phytochemical compositions among cultivars. It offered a comprehensive view for leaf metabolomes of Wuyi Rock tea cultivars in general and provided basis for future characterizations of nutritional values, sensory qualities and biological properties of Wuyi Rock tea.

## 2. Results and Discussion

### 2.1. Major Tea Leaf Metabolites Showed both Universal and Cultivar-Dependent Accumulation Patterns

**Figure 1:**
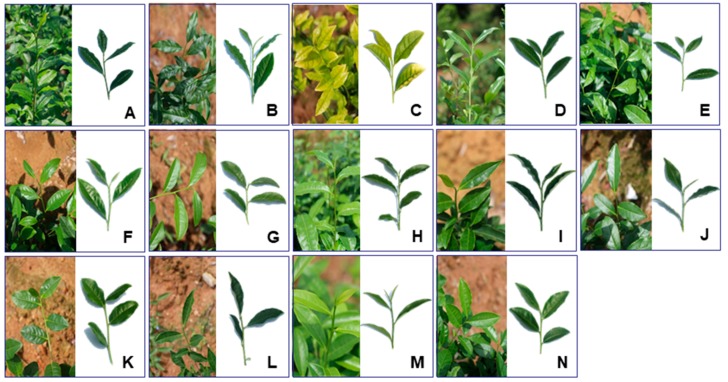
Leaf phenotypes of 14 Wuyi Rock tea cultivars. (**A**) ‘Dahongpao’ (DHP); (**B**) ‘Tieluohan’ (TLH); (**C**) ‘Baijiguan’ (BJG); (**D**) ‘Shuijingui’ (SJG); (**E**) ‘Bantianyao’ (BTY); (**F**) ‘Shuixian’ (SX); (**G**) ‘Rougui’ (RG); (**H**) ‘Beidou’ (BD); (**I**) ‘Queshe’ (QS); (**J**) ‘Xiaoyemaoxie’ (XYMX); (**K**) ‘Jinfenghuang’ (JFH); (**L**) ‘Aijiaowulong’ (AJWL); (**M**) ‘Guazijin’ (GZJ); (**N**) ‘Jinsuoshi’ (JSS).

**Figure 2:**
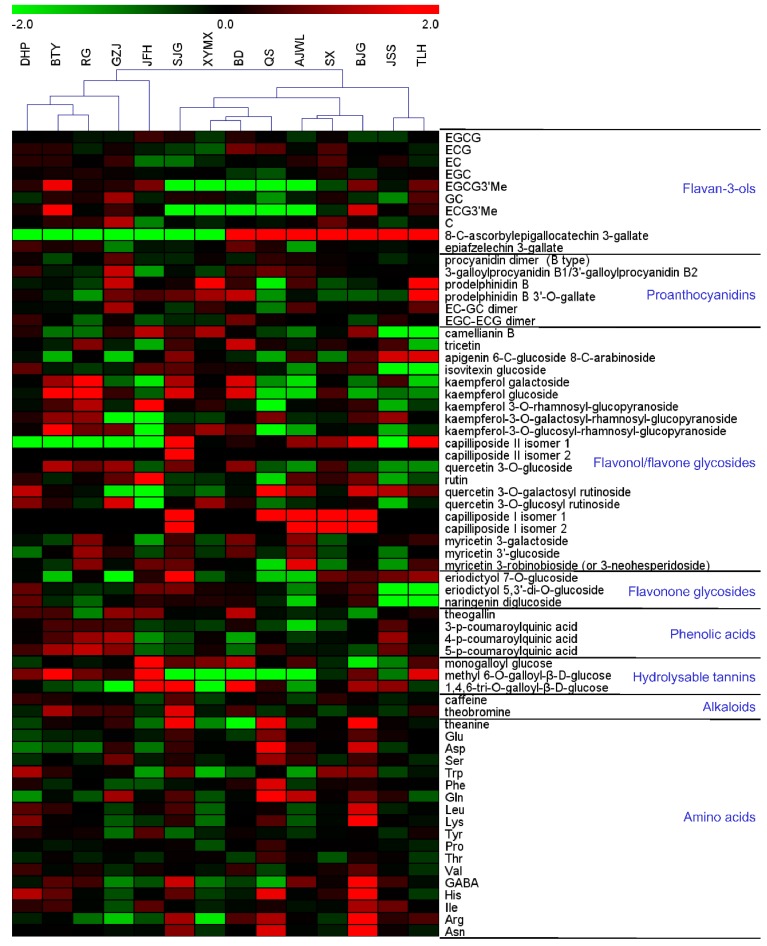
Comparisons of metabolite levels in 14 tea cultivars. The analysis is based on the normalized average signal abundance from three biological replicates for each cultivar. Normalized values are shown on a color scale proportional to the content of each metabolite and are expressed as log2 using the MultiExperiment Viewer software (MeV v4.9.0, J. Craig Venter Institute, La Jolla, CA, USA).

To identify abundant metabolites and assess metabolite differences in 14 Wuyi Rock cultivars, which included ‘Dahongpao’ (DHP), ‘Tieluohan’ (TLH), ‘Baijiguan’ (BJG), ‘Shuijingui’ (SJG), ‘Bantianyao’ (BTY), ‘Shuixian’ (SX), ‘Rougui’ (RG), ‘Beidou’ (BD), ‘Queshe’ (QS), ‘Xiaoyemaoxie’ (XYMX), ‘Jinfenghuang’ (JFH), ‘Aijiaowulong’ (AJWL), ‘Guazijin’ (GZJ) and ‘Jinsuoshi’ (JSS) (Figure 1), non-targeted analysis based on UPLC-QTOF MS was performed to profile tea leaves. Forty-nine major metabolites were tentatively assigned based on their accurate masses, MS/MS fragmentation patterns and UV absorbance, in comparison to standard compounds and references (Table 1). Catechins, caffeine and free amino acids have been shown in a large body of literatures to contribute significantly to the taste and flavor quality of tea [6,18,19,20]. Therefore, absolute quantifications of these compounds, along with rutin, were carried out using UPLC-QqQ MS to enable comparisons with tea cultivars from other studies. The quantification results were shown in Table 2. Relative differences in the metabolites found in each sample were depicted in a heat map, which integrated measurements from both non-targeted and targeted analyses (Figure 2 and Figure S1).

Most compounds were detected in all tea cultivars suggesting the presence of common machinery for secondary metabolism in tea plants (Figure 2). However, sharp variations between cultivars (VIP > 1 and *p* < 0.05) were found for many metabolite classes, such as flavan-3-ols, proanthocyanidins, flavonol glycosides, flavone glycosides, flavonone glycosides, phenolic acid derivatives, hydrolysable tannins, alkaloids and amino acids (Figure 2).

**Figure 3:**
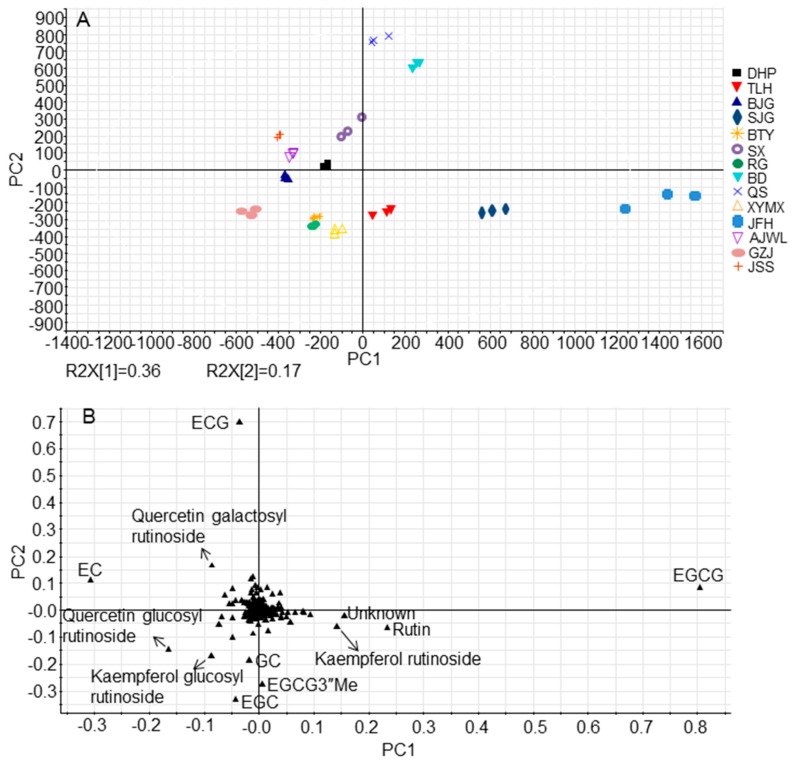
Principal component analysis (PCA) of methanol extracts of tea leaves. (**A**) Score plot of PCA demonstrating differences in metabolite profiles between leaf samples based on 466 filtered single molecular features detected by UPLC-QTOF MS in ESI^−^. The principal components 1 and 2 explained 36.0% and 17.0% of total variance, respectively. For each cultivar, three biological replicates were prepared, where one replicate was a pool of 7–8 tea leaves. R2X, explained variation; (**B**) Loading plot of PCA indicating primary differential metabolites.

In PCA score plot (Figure 3A), the first and the second principal components explained 36.0% and 17.0% of the variation, respectively. Samples were clustered mainly according to their biological replicates in the PCA score plot, except that cultivars RG and BTY showed close aggregation, indicating inter-cultivar variations in metabolite profiles of Wuyi Rock tea cultivars. In addition, cultivars JFH and SJG were clearly separated from other cultivars along PC1 whereas cultivars QS and BD were separated from others along PC2. As these cultivars were grown in the same geographic region under the same cultivation condition, influences of varying environmental conditions on the chemical make-ups of tea leaves could be minimized. As a result, differences in metabolite compositions were largely attributed to particular genotype traits.

To investigate major differential metabolites, a PCA loading plot was applied (Figure 3B). The major groups that stood out in the plot corresponded to the MS signals of catechins (e.g., (−)-epigallocatechingallate (EGCG), (−)-epicatechingallate (ECG), (−)-epigallocatechin (EGC), (−)-epicatechin (EC), (−)-gallocatechin (GC), and (−)-epigallocatechin 3-(3-*O*-methylgallate) (EGCG3″Me)) and flavonol glycosides (e.g., rutin, quercetin galactosyl rutinoside, quercetin glucosyl rutinoside, kaempferol rutinoside and kaempferol glucosyl rutinoside). This inferred that catechins as well as quercetin and kaempferol derivatives were the most critical parameters for cultivar discrimination.

### 2.2. Flavan-3-ols Exhibited Variable Levels in Tea Leaves

A total of 10 flavan-3-ols was tentatively identified by UPLC-QTOF MS, including GC, EGC, (−)-catechin (C), EC, EGCG, 8-*C*-ascorbylepigallocatechin 3-gallate, EGCG3″Me, ECG, epicatechin 3-(3-*O*-methylgallate) (ECG3″Me) and epiafzelechin 3-gallate (Table 1). Major flavan-3-ols included EGCG, EGC, EC and ECG, which occurred at descending levels in all cultivars examined (Table 2). In general, phenolic contents of tea cultivars applied to black and oolong tea are higher than that of green tea [7]. EGCG, as the most dominant flavan-3-ol, ranged from 66.93 mg/g in cultivar BJG to 128.08 mg/g in cultivar JFH, whereas GC (1.44–6.75 mg/g) and C (0.37–1.76 mg/g) were only minor components (Table 2).

EGCG3″Me, an *O*-methylated catechin, was detected in all tea cultivars, albeit in very low abundance in cultivars SJG, BD, QS, XYMX and AJWL (Figure 2 and Table 2). Methylated catechins have attracted much attention because of their stronger anti-allergic activities than catechins, including EGCG [29,30]. Efforts have been made to screen different tea varieties to identify cultivars enriched in EGCG3″Me [6,31]. Lv and coworkers identified four out of 71 Chinese tea cultivars with EGCG3″Me contents higher than 10 mg/g; interestingly, they are all oolong tea cultivars, implying that oolong tea cultivars may be a good source for finding EGCG3″Me-rich tea cultivars [31]. Supporting this notion, we found that cultivar BTY contained the highest content of EGCG3″Me (12.05 mg/g) (Table 2). Moreover, the other three cultivars, TLH, BJG and JFH, also produced medium levels of EGCG3″Me (≥6 mg/g). The distribution of a second *O*-methylated catechin, ECG3″Me, which also exhibited a strong anti-inflammatory activity in vitro [29], resembled that of EGCG3″Me, ranging from barely detectable in cultivars SJG, BD, QS, XYMX and AJWL to being highest in cultivar BTY. Due to the lack of an authentic standard, the absolute quantification of ECG3″Me was impossible. Nevertheless, the level of this compound somewhat demonstrated a positive correlation with the EGCG3″Me level in tea cultivars (Figure 2 and Figure S1). As a result, there is potentially a higher chance of finding tea cultivars rich in ECG3″Me among cultivars rich in EGCG3″Me.

An ascorbic acid-appended EGCG derivative, namely, 8-*C*-ascorbylepigallocatechin 3-gallate, was another interesting flavan-3-ol present as a minor constituent in seven cultivars, TLH, BJG, SX, BD, QS, AJWL and JSS (Figure 2). Initially isolated from a commercial oolong tea sample, this compound was structurally elucidated through NMR spectroscopy by Hashimoto and coworkers [32]. Subsequent activity tests showed that it demonstrated inhibitory effects against HIV replication in H9 lymphocyte cells [33] and pancreatic lipase [34]. Information on the distribution of 8-*C*-ascorbylepigallocatechin 3-gallate among tea cultivars is scarce. Nonetheless, considering where this compound was first isolated and its high occurrence in the current study, Wuyi Rock tea cultivars may be a promising source for compound isolation to further explore its therapeutic potential. 

### 2.3. Cultivar JFH Possessed High Contents of Rutin and Kaempferol Rutinoside

Flavonol glycosides (FOGs) are one of most important phenolic compounds in tea besides catechins. Though less abundant than catechins, they confer velvety and astringent tastes to tea infusions at much lower thresholds and hence are key tea taste determinants [35]. Unambiguous FOG assignments are difficult due to the fact that many authentic standards of FOGs in tea are not commercially available. Moreover, FOGs usually contain several positional isomers. Nevertheless, galactosyl flavonols were reported to elute earlier than glucosyl flavonols [15]. Taking account of the differences in chromatographic retention behaviors, in combined with analyses of MS^2^ fragmentation patterns and UV absorbance (if available), we tentatively identified 18 FOGs in the current study. These FOGs, most commonly kaempferol and quercetin derivatives, were mainly present in the form of mono-, di-, tri- and tetraglycosides (Table 1). Many FOGs have been previously detected in processed tea products or fresh tea leaves [2,36]. 

**Figure 4:**
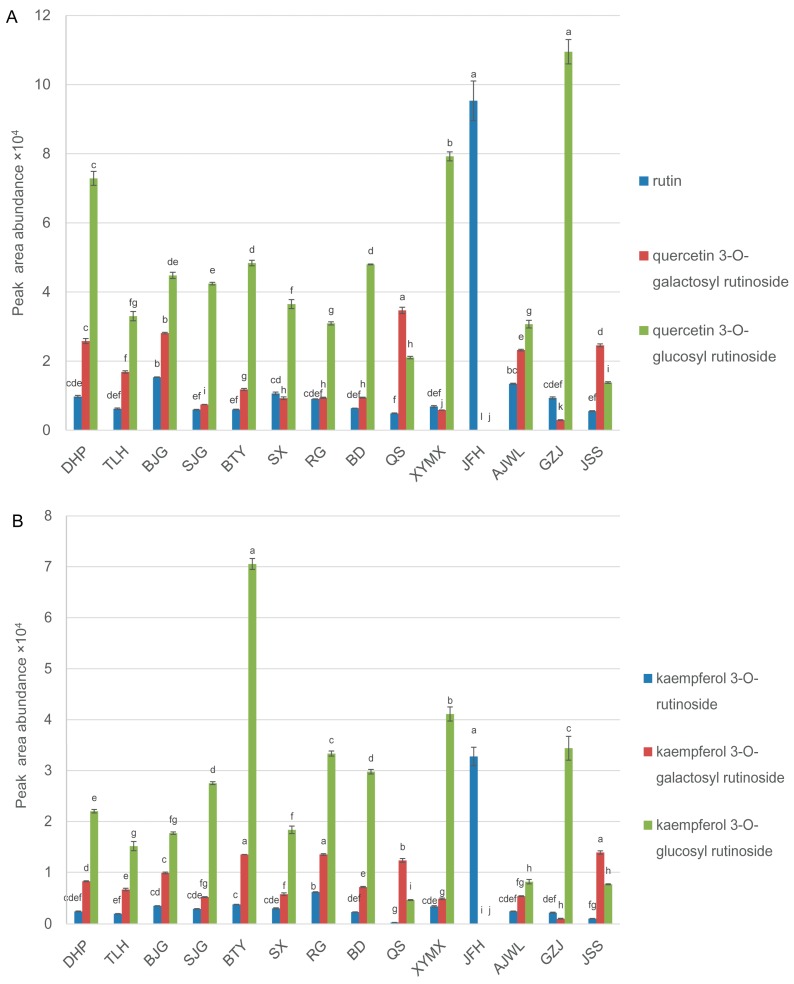
Mean peak area abundance values (±SD) of (**A**) some quercetin glycosides and (**B**) some kaempferol glycosides in leaves of 14 tea cultivars. Different letters on top of the vertical bars of the graph indicate significant differences among the samples, which were determined by Tukey’s HSD test at *p* < 0.05.

Contents of kaempferol and quercetin glycosides varied widely between tea cultivars (Figure 2). In particular, the rutin (also called quercetin 3-*O*-rutinoside) content in cultivar JFH (5.40 mg/g) was found to be significantly higher (*p* < 0.01) than in other cultivars (between 0.18–0.89 mg/g) (Figure 4A and Table 2). Apart from rutin, the highest level of kaempferol 3-*O*-rutinoside was also detected in cultivar JFH (Figure 4B). In contrast, four flavonol triglycosides, kaempferol 3-*O*-galactosyl rutinoside, kaempferol 3-*O*-glucosyl rutinoside, quercetin 3-*O*-galactosyl-rutinoside and quercetin 3-*O*-glucosyl rutinoside, were barely detectable in this cultivar (Figure 4A,B). Quercetin 3-*O*-galactosyl rutinoside and quercetin 3-*O*-glucosyl rutinoside could be synthesized from rutin by glycosyltransferases. Kaempferol 3-*O*-galactosyl rutinoside and kaempferol 3-*O*-glucosyl rutinoside may derive from kaempferol 3-*O*-rutinoside catalyzed by the same type of enzymes. In cultivar JFH, we speculate that enzyme(s) responsible for glycosylation of flavonol diglycosides are either not functional or expressed at very low levels, accounting for the high accumulations of rutin and kaempferol 3-*O*-rutinoside. Alternatively, genes which are critical for flavonoid metabolism and catabolism in cultivar JFH may be differentially regulated. Comparing gene expressions in the phenylpropanoid pathway among these cultivars would further shed light on flavonoid biosynthesis in tea. A number of pharmacological properties of rutin, such as anti-inflammation, anti-microbial, anti-tumor and anti-asthma, have been well documented [37]. Therefore, finding tea cultivars with high yields of flavonoids such as rutin could be useful in diversifying the utilization of functional components in tea resources.

### 2.4. Cultivars BJG, SJG and QS Demonstrated High Levels of Amino Acids in Leaves

Amino acid constituents of tea leaves have a large impact on the taste and aroma properties of processed tea [18]. There exists a positive correlation between the quality of tea and amino acid contents [18]. Theanine, glutamate and serine are key components imparting “umami” or “brothy” taste to tea infusions [19,38]. To compare amino acid profiles between leaves of different cultivars, hydrophilic interaction liquid chromatography (HILIC) tandem mass spectrometry was applied since amino acids are typically not well resolved in C18 columns. In total, 18 amino acids in varying concentrations were detected (Table 2). Theanine, a non-proteinogenic amino acid synthesized from glutamate and ethylamine by theanine synthetase, has been shown in many studies as the most abundant free amino acid in tea plants [20,39,40,41]. As expected, theanine was found to be the most predominant free amino acid in 13 out of 14 cultivars examined, accounting for 42.3–72.3% of total free amino acids in leaves. Other abundant amino acids included glutamate, aspartate and serine (Table 2). In contrast, cultivar BD contained a higher level (*p* < 0.01) of glutamate (1.2 mg/g) than theanine (0.8 mg/g). Moreover, the total amino acid content (3.11 mg/g) in leaves of cultivar BD was lowest. Cultivars BJG, SJG and QS were characterized by high levels of amino acids (Table 2). Not only containing markedly higher (*p* < 0.05) amounts of theanine (14.32, 12.08 and 11.63 mg/g for cultivars BJG, SJG and QS, respectively), they also demonstrated high accumulations of other amino acids, suggesting an overall up regulation of amino acid metabolism. For example, glutamate and aspartate were at high levels in both cultivars. The glutamine level in cultivar QS and the arginine level in cultivar BJG were significantly higher (*p* < 0.01) than other cultivars.

Interestingly, cultivar BJG is a light-sensitive albino tea variety (Figure 1C) [42]. A recent study showed that cultivar BJG exhibited yellow leaf phenotype, and reduced synthesis of chlorophyll and carotenoid under high light intensity but could turn green when transferred to low intensity light [42]. Similar scenarios were described for other albino tea cultivars such as ‘Anji Baicha’, whose albinism is induced by temperature instead of light [43]. Feng and coworkers reported that theanine and glutamate levels in some temperature-sensitive albino tea cultivars were higher than in normal green cultivars [19]. Similar to temperature-sensitive cultivars, cultivar BJG also contained the highest level of theanine and a relatively higher level of glutamate, second only to cultivar QS (Table 2). One possible explanation is that suppressed chlorophyll biosynthesis leads to elevated levels of glutamate, which provides more substrates for theanine synthesis [43]. 

### 2.5. Purine Alkaloids Exhibited Variable Levels in Tea Leaves

Purine alkaloids are naturally found in tea. The biosynthesis of purine alkaloids, mainly caffeine and theobromine, has been extensively investigated in tea plants [41,44,45]. The caffeine is the most abundant purine alkaloid in tea leaves, ranging between 1.5–5% [36]. Among the 14 cultivars, the caffeine content in leaves was found to vary between 1.38% (cultivar JFH) and 2.98% (cultivar SJG) (Table 2), consistent with the previous report [36]. Moreover, cultivar SJG also had markedly a higher (*p* < 0.05) content of theobromine than other cultivars, followed by cultivar BTY (Figure 2). Previous studies suggested that genotypic factors other than environmental factors may have more effects on the caffeine content [46]. Therefore, differences in the abundances of purine alkaloids in the current study may be the result of genetic variations.

## 3. Materials and Methods 

### 3.1. Chemicals 

(−)-Epigallocatechingallate, (−)-epigallocatechin, (−)-catechin, (−)-epicatechingallate, (−)-epicatechin, (−)-gallocatechin, rutin and L-theanine (all with purity≥95%) were obtained from Sigma-Aldrich (St. Louis, MO, USA). (−)-Epigallocatechin3-(3-*O*-methylgallate) (≥95%) and kaempferol 3-rutinoside (≥98%) were purchased from ChemFaces (Wuhan, China). Caffeine (≥98%) was obtained from Yuanye Biotechnology Inc. (Shanghai, China). Theobromine (≥99%) and kaempferol glucoside (≥98%) were obtained from BioBioPha Co., Ltd. (Kunming, China). Theogallin (≥95%) was kindly provided by Dr. Qingxie Chen of Fujian Agriculture and Forestry University, China. Acetonitrile (MS grade), methanol (HPLC grade) and formic acid (98%) were obtained from Sigma-Aldrich. Deionized water was produced by a Milli-Q water purification system (Millipore, Billerica, MA, USA). 

### 3.2. Tea Samples and Sample Preparation 

All tea plants of *Camellia sinensis* (five-year-old) used in the current study were grown at the same tea germplasm garden and under the same cultivation practice, which was managed by the Wuyi Star Tea Industry Co., Ltd., Wuyishan City, Fujian, China (latitude: 27.71° N, longitude: 118.00° E). The fully expanded second leaves were collected from 14 Wuyi Rock tea cultivars on 9 May 2015. The cultivars included ‘Dahongpao’, ‘Tieluohan’, ‘Baijiguan’, ‘Shuijingui’, ‘Bantianyao’, ‘Shuixian’, ‘Rougui’, ‘Beidou’, ‘Queshe’, ‘Xiaoyemaoxie’, ‘Jinfenghuang’, ‘Aijiaowulong’, ‘Guazijin’ and ‘Jinsuoshi’ (Figure 1). For each cultivar, three biological replicates were collected with each replicate gathered from 7–8 individual tea plants. The excised leaf samples were immediately frozen in liquid nitrogen, brought back to lab and stored at −80 °C until analysis. 

Extraction of tea leaves were carried out as previously described with some minor modifications [47]. Briefly, frozen tea leaves were individually ground to fine powders using precooled mortars and pestles. Following lyophilization, 30 mg (± 0.5 mg) of ground samples was weighted and 1.2 mL of 70% (*v*/*v*) methanol was added for metabolite extraction. Samples were vortexed, sonicated at 25 °C for 20 min and centrifuged (10 min, 12,000 *g*). Supernatants were diluted 50-fold with 70% (*v*/*v*) methanol, filtered through a 0.22 μm PVDF filter (Millipore) and stored at −20 °C until analyzed. Three biological sample replicates were prepared for each cultivar.

### 3.3. UPLC-QTOF MS-Based Non-Targeted Metabolite Analysis

Aliquots (1 µL) of above extracts were analyzed on a Waters Acquity UPLC system coupled in tandem to a Waters photodiode array (PDA) detector and a SYNAPT G2-Si HDMS QTOF mass spectrometer (Waters, Manchester, UK). Chromatographic separation was performed on a Waters Acquity UPLC HSS T3 column (2.1 × 100 mm, 1.8 µm) at 40 °C with water containing 0.1% formic acid (phase A) and acetonitrile containing 0.1% formic acid (phase B) for chromatographic elution: 0–2 min (99–93% A), 2–13 min (93–60% A), 13–14 min (60–1% A) and 14–17 min (1–1% A). The flow rate was set at 0.3 mL/min. 

Samples were run in both positive and negative ionization modes as separate chromatographic runs. Following settings were applied during LC-MS runs: capillary voltage, 2.0 kV (ESI^+^) and 2.5 kV (ESI^−^); cone voltage, 40 eV; collision energy, 4 eV; source temperature, 120 °C; desolvation temperature, 500 °C; cone gas flow, 50 L/h; desolvation gas flow, 800 L/h; *m*/*z* range, 50–1200 Da. The collision energy ramp for MS^e^ (continuum mode) was set from 10 to 50 eV. LockSpray (leucine encephalin) reference ions with *m*/*z* of 556.2771 (for ESI^+^) or 554.2615 (for ESI^−^) were infused during data acquisition for online calibration. Each triplicate tea sample was analyzed once. 

Quality control (QC) samples were prepared by mixing 30 mg of one leaf sample to become a combined sample. QC samples were injected throughout the analytical runs (every five samples) to check the instrument performance. The MassLynx software (version 4.1, Waters, Milford, MA, USA) was used to control all instruments and calculate accurate masses. 

### 3.4. UPLC-QqQ MS-Based Targeted Quantification of Catechins, Rutin, Caffeine and Amino Acids

For quantifications of catechins, rutin, caffeine and amino acids, 2 µL of above extracts, with appropriate dilutions within the range of the calibration curve, were injected on a Waters Acquity UPLC system coupled in tandem to a Waters photodiode array (PDA) detector and a XEVO TQ-S MS triple quadrupole mass spectrometer (Waters, Milford, MA, USA). 

To detect catechins, rutin and caffeine, chromatographic separation was achieved on a Waters Acquity UPLC BEH C18 column (2.1 × 100 mm, 1.7 µm) at 40 °C with water containing 0.1% formic acid (phase A) and acetonitrile containing 0.1% formic acid (phase B) for chromatographic elution: 0–12 min (95–83% A), 12–13 min (83–0% A) and 13–16.5 min (0–0% A). The flow rate was set at 0.3 mL/min. Mass spectrometry was performed in the ESI^−^ mode for catechins and rutin, and in the ESI^+^ mode for caffeine with the following settings: capillary voltage: 3.0 kV (ESI^+^) and 2.0 kV (ESI^−^); desolvation temperature: 400 °C; source temperature: 150 °C; cone gas flow: 150 L/h; desolvation gas flow: 800 L/h. Collision energy and cone voltage were optimized for above compounds with multiple reaction monitoring (MRM) for quantification. Calibration curves generated by injecting increasing concentrations of chemical standards were used to determine the absolute concentrations of catechins, rutin and caffeine. 

Amino acids were measured in the same manner except that the chromatographic separation was achieved on a Merck SeQuant ZIC-HILIC column (2.1 × 100 mm, 5 µm) at 40 °C with water containing 5 mM ammonium acetate (phase A) and acetonitrile containing 0.1% formic acid (phase B) for chromatographic elution: 0–13 min (5–41% A), 13–15 min (41–60% A) and 15–20 min (60–5% A). The flow rate was set at 0.4 mL/min. Mass spectrometry was performed in the ESI^+^ mode using the same setting for caffeine. In all cases, the MassLynx software (version 4.1, Waters, Milford, MA, USA) was used for instrument control and data acquisition. Each triplicate tea sample was analyzed once.

### 3.5. Data Processing and Statistical Analysis

Resulting chromatograms from UPLC-QTOFMS were processed using Progenesis QI software (version 2.1, Nonlinear Dynamics, New Castle upon Tyne, UK) with default settings for peak picking, normalization (normalized to all compounds), signal integration and initial peak assignments. Only chromatograms between elution time 1–14 min were included in the analysis. The final data set contained 1550 molecular features in the ESI^−^ mode and 992 molecular features in the ESI^+^ mode. For comparing the abundances of molecular features, the data matrix consisting of mass features (including retention time and accurate mass values) and peak area values was exported from Progenesis QI to Excel. The mean peak area abundance values from three biological replicates of the same cultivar were calculated and differences in metabolite signal abundances were compared across cultivars. 

The raw 1550 molecular features detected in the ESI^−^ mode were filtered to only include single features. After filtering, the remaining 466 molecular features were fed into PCA analysis to observe intrinsic metabolite variance between cultivars; PLS-DA was performed to identify differential metabolites using Progenesis QI extension EZinfo after Pareto scaling. One-way ANOVA was used to measure the significance of metabolites in cultivar discrimination using SPSS (version 13.0, SPSS, Chicago, IL, USA). Significantly different metabolites between samples were selected with variable importance in the projection (VIP) > 1 and a *p* value < 0.05. Heat map with hierarchical clustering (Pearson’s correlation, average linkage) was generated with MultiExperiment Viewer software (version 4.9.0, J. Craig Venter Institute, La Jolla, CA, USA) to visualize accumulation patterns of annotated major metabolites between sample types. Before analysis, the data were log2 transformed and normalized to the median level of individual compounds, combining data from metabolite analyses by UPLC-QTOF MS and UPLC-QqQ MS. The data matrix used for PCA and PLS-DA analyses was listed in Table S1. 

### 3.6. Metabolite Identification 

Annotation obtained from Progenesis QI was used as a starting point for manual peak identification. Metabolites were identified by comparing accurate masses, MS/MS fragmentation patterns and isotope patterns with authentic standards, online metabolite databases of Metlin [21], HMDB [48], MassBank [49], ReSpect [22], KNApSAcK [50] and literature references [2,11,27,28,51]. Each mass spectrum was manually inspected to verify whether software-predicted fragments were derived from a single metabolite. UV spectra (Waters, Milford, MA, USA) were used for identification whenever possible.

## 4. Conclusions

To the best of our knowledge, this is the first exhaustive study of leaf metabolite profiles of different Wuyi Rock tea cultivars. A combined UPLC-QTOF MS and UPLC-QqQ MS approach coupled with multivariate data analysis revealed fundamental varietal differences in primary and secondary metabolism between cultivars. Those differential metabolites mainly include phenolic compounds (e.g., flavan-3-ols, flavonol glycosides and phenolic acids), alkaloids and amino acids. Major catechins as well as quercetin and kaempferol glycosides were determined as critical for cultivar discrimination. The functional compounds found in leaves of Wuyi Rock tea cultivars as well as the knowledge on the cultivar-specific differences provides insights for their potential applications as dietary supplements or nutraceuticals. For instance, cultivar BTY would be an excellent target for anti-allergic study owing to the production of high levels of methylated catechins. Cultivar JFH may serve as a stable source for rutin and kaempferol rutinoside. Cultivars BJG, SJG and QS could be explored as a prominent source for theanine. The metabolites identified in the current study could potentially be used as chemical markers for tea plant fingerprinting, cultivar identification and tea authentication. It also provides valuable information to tea breeders in selecting breeding materials with desirable traits. On the other hand, chemical constituents of processed tea are influenced by both cultivars and processing techniques. Studies by other research groups have shown that both volatile and non-volatile compounds undergo substantial changes during the manufacture of oolong tea [1,4,52].Therefore, whether and how potential markers uncovered in the current study could be applied for analyzing processed Wuyi Rock tea samples warrants further investigations.

## Figures and Tables

**Table 1:** Metabolites putatively identified in 14 tea cultivars by UPLC-QTOFMS.

Compound#	Tentative Assignments	RT (min)	Detected [M − H]^−^ (*m*/*z*)	Theoretical [M − H]^−^ (*m*/*z*)	Mass Error (ppm)	Formula	MS/MS Fragments	Ref.
**Catechins**								
**1**	GC	3.84	305.0668	305.0661	0.51	C_15_H_14_O_7_	219.0660, 179.0348, 167.0347, 139.0397, 125.0242	Authentic standard ^b^
**2**	EGC	4.93	305.0680	305.0661	1.04	C_15_H_14_O_7_	219.0663, 179.0350, 167.0349, 139.0400, 125.0245	Authentic standard ^b^
**3**	C	5.36	289.0718	289.0712	0.20	C_15_H_14_O_6_	245.0817, 203.0710, 125.0242	Authentic standard ^b^
**4**	EC	6.28	289.0722	289.0712	2.13	C_15_H_14_O_6_	245.0820, 203.0711, 123.0450	Authentic standard ^b^
**5**	EGCG	6.35	457.0783	457.0771	2.60	C_22_H_18_O_11_	305.0662, 169.0143, 125.0244	Authentic standard ^b^
**6**	8-*C*-ascorbylepigallocatechin 3-gallate	6.65	631.0938	631.0935	-0.37	C_28_H_24_O_17_	479.0821, 316.0218	[21]
**7**	EGCG3″Me	7.42	471.0933	471.0927	0.16	C_23_H_20_O_11_	305.0667, 287.0560, 183.0300, 161.0243	Authentic standard ^b^
**8**	ECG	7.86	441.0827	441.0822	0.51	C_22_H_18_O_10_	331.0458, 289.0719, 245.0818, 169.0145, 125.0245	Authentic standard ^b^
**9**	ECG3″Me	8.92	455.0984	455.0978	-0.02	C_23_H_20_O_10_	289.0717, 183.0298	Authentic standard ^b^
**10**	epiafzelechin 3-gallate	8.97	425.0880	425.0873	0.41	C_22_H_18_O_9_	273.0765, 255.0661	[21]
**Proanthocyanidins**								
**11**	prodelphinidin B	4.11	609.1251	609.1244	0.13	C_30_H_26_O_14_	441.0825, 423.0718, 305.0667, 125.0243	[2]
**12**	EC-GC dimer	4.80	593.1301	593.1295	0.06	C_30_H_26_O_13_	423.0714, 305.0659, 289.0717	[21]
**13**	prodelphinidin B2 (or B4) 3′-*O*-gallate	5.12	761.1357	761.1354	-0.27	C_37_H_30_O_18_	609.1236, 591.1144, 577.1347, 423.0717	[2]
**14**	procyanidin dimer (B type)	5.68	577.1352	577.1346	0.02	C_30_H_26_O_12_	451.1029, 425.0874, 407.0768, 289.0716	[22]
**15**	EGC-ECG dimer	6.04	745.1409	745.1405	-0.19	C_37_H_30_O_17_	593.1298, 423.0714, 407.0768, 177.0191	[23]
**16**	3-galloylprocyanidin B1/3′-galloylprocyanidin B2	6.78	729.1458	729.1456	-0.36	C_37_H_30_O_16_	407.0766, 289.0716	[23]
**Flavonol/flavone glycosides**								
**17**	isovitexin glucoside	6.08	595.1655 ^a^	595.1663 ^a^	-0.40	C_27_H_30_O_15_	313.0711	[24]
**18**	apigenin 6-*C*-glucoside 8-*C*-arabinoside	6.91	563.1405	563.1401	-0.22	C_26_H_28_O_14_	473.1086, 443.0980, 383.0769, 353.0664	[24]
**19**	myricetin 3-robinobioside (or 3-neohesperidoside)	6.93	625.1407	625.1405	-0.44	C_27_H_30_O_17_	316.0219	[21]
**20**	myricetin 3-galactoside	7.02	479.0829	479.0826	0.36	C_21_H_20_O_13_	316.0223, 315.0141, 271.0245	[25]
**21**	myricetin 3′-glucoside	7.12	479.0830	479.0826	-0.22	C_21_H_20_O_13_	316.0224, 315.0146, 271.0245	[25]
**22**	quercetin 3-*O*-galactosyl rutinoside	7.21	771.1990	771.1984	0.03	C_33_H_40_O_21_	609.1434, 463.0903, 301.0339, 300.0266	[11]
**23**	quercetin 3-*O*-glucosyl rutinoside	7.36	771.1991	771.1984	0.23	C_33_H_40_O_21_	609.1458, 301.0348, 300.0272	[11]
**24**	camellianin B	7.69	579.1704 ^a^	579.1714 ^a^	-0.75	C_27_H_30_O_14_	433.1129, 313.0709	[26]
**25**	rutin	7.70	609.1455	609.1456	-0.93	C_27_H_30_O_16_	300.0274, 299.0195	Authentic standard ^b^
**26**	kaempferol-3-*O*-galactosylrutinoside	7.72	755.2040	755.2035	-0.05	C_33_H_40_O_20_	533.1294, 285.0398, 284.0319	[2]
**27**	tricetin	7.90	303.0504^a^	303.0505 ^a^	1.56	C_15_H_10_O_7_	285.0402, 257.0450	[21]
**28**	kaempferol-3-*O*-glucosylrutinoside	8.00	755.2042	755.2035	0.19	C_33_H_40_O_20_	593.1511, 285.0403, 284.0325	[2]
**29**	quercetin 3-*O*-glucoside	8.02	463.0879	463.0877	-0.62	C_21_H_20_O_12_	300.0274, 299.0195, 243.0297	[25]
**30**	kaempferol 3-*O*-rutinoside	8.43	593.1511	593.1506	-0.21	C_27_H_30_O_15_	501.0102, 285.0399, 284.0326	Authentic standard ^b^
**31**	kaempferol galactoside	8.51	447.0929	447.0927	-0.84	C_21_H_20_O_11_	285.0387, 284.0317	
**32**	kaempferol glucoside	8.78	447.0929	447.0927	-0.76	C_21_H_20_O_11_	284.0324, 255.0295, 227.0349	Authentic standard ^b^
**33**	capilliposide I isomer 1	9.94	1065.3052 ^a^	1065.3087 ^a^	-2.68	C_48_H_56_O_27_	617.2078, 449.1078, 303.0506	[27]
**34**	capilliposide II isomer 1	10.19	1049.3113 ^a^	1049.3138 ^a^	-1.84	C_48_H_56_O_26_	741.2036, 595.1495, 287.0553	[27]
**35**	capilliposide I isomer 2	10.60	1065.3061 ^a^	1065.3087 ^a^	-1.97	C_48_H_56_O_27_	617.2083, 449.1086, 303.0514	[27]
**36**	capilliposide II isomer 2	10.88	1049.3114 ^a^	1049.3138 ^a^	-1.78	C_48_H_56_O_26_	741.2048, 287.0564	[27]
**Flavonone glycosides**								
**37**	eriodictyol 5,3′-di-*O*-glucoside	6.08	611.1617	611.1612	-0.10	C_27_H_32_O_16_	491.1189, 449.1292, 329.0869	[21]
**38**	naringenin diglucoside	6.16	595.1664	595.1663	-0.78	C_27_H_32_O_15_	577.1552, 475.1243, 433.1348, 381.0827, 313.0923	[21]
**39**	eriodictyol 7-*O*-glucoside	6.57	449.1086	449.1084	-0.68	C_21_H_22_O_11_	329.0657, 197.0455	[21]
**Phenolic acids**								
**40**	theogallin	2.90	343.0669	343.0665	0.16	C_14_H_16_O_10_	191.0560	Authentic standard ^b^
**41**	3-*p*-coumaroylquinic acid	5.18	337.0928	337.0923	-0.25	C_16_H_18_O_8_	163.0399	[2]
**42**	4-*p*-coumaroylquinic acid	6.15	337.0924	337.0923	-0.36	C_16_H_18_O_8_	191.0542, 173.0454, 163.0398,119.0500,111.0441, 93.0343	[2]
**43**	5-*p*-coumaroylquinic acid	6.42	337.0925	337.0923	-0.08	C_16_H_18_O_8_	173.0457, 163.0396, 119.0499, 93.0343	[2]
**Hydrolysable tannins**								
**44**	monogalloyl glucose	2.44	331.0668	331.0665	-0.80	C_13_H_16_O_10_	271.0454, 211.0247, 169.0140, 125.0242	[28]
**45**	methyl 6-*O*-galloyl-*β*-d-glucose	3.67	345.0823	345.0822	-1.09	C_14_H_18_O_10_	285.0611, 225.0401, 183.0296	[21]
**46**	1,4,6-tri-*O*-galloyl-*β*-d-glucose	6.64	635.0894	635.0884	1.60	C_27_H_24_O_18_	483.0777, 465.0666, 423.0524, 313.0562, 241.0348, 169.0142, 125.0236	[25]
**Alkaloids**								
**47**	theobromine	3.80	181.0725 ^a^	181.0726 ^a^	3.53	C_7_H_8_N_4_O_2_	138.0671	Authentic standard ^b^
**48**	caffeine	5.60	195.0893 ^a^	195.0882 ^a^	5.60	C_8_H_10_N_4_O_2_	138.0670	Authentic standard ^b^
**Amino acids**								
**49**	theanine	1.43	175.1085 ^a^	175.1083 ^a^	4.72	C_7_H_14_N_2_O_3_	158.0823, 129.1030	Authentic standard ^b^

^a^ [M + H]^+^. ^b^ This letter indicates that identification of the compound was confirmed by the authentic standard.

**Table 2:** Abundance (mg/g DW) of catechins, rutin, caffeine and amino acids in tea leaves in relation to cultivars.

Compound	DHP	TLH	BJG	SJG	BTY	SX	RG	BD	QS	XYMX	JFH	AJWL	GZJ	JSS
**Catechins**	**210.09 ± 12.55 bc**	**201.42 ± 5.35 c**	**155.29 ± 6.42 e**	**199.58 ± 4.77 cd**	**225.73 ± 6.42 ab**	**233.28 ± 4.11 a**	**195.99 ± 8.44 cd**	**210.14 ± 16.03 abc**	**177.60 ± 2.85 de**	**162.51 ± 5.88 e**	**218.92 ± 3.72 abc**	**167.86 ± 4.68 e**	**201.94 ± 9.50 c**	**165.06 ± 6.09 e**
EGCG	94.67 ± 5.17 d	95.07 ± 2.93 d	66.93 ± 3.97 f	111.71 ± 1.76 bc	97.81 ± 2.50 d	103.73 ± 1.66 cd	81.87 ± 3.43 e	116.21 ± 8.70 b	96.45 ± 2.08 d	69.71 ± 2.25 f	128.08 ± 1.63 a	71.20 ± 2.23 ef	81.60 ± 3.61 e	68.27 ± 3.35 f
ECG	22.83 ± 1.56 c	15.17 ± 0.56 fg	18.13 ± 0.93 de	13.09 ± 0.32 gh	22.83 ± 0.60 c	26.51 ± 0.26 b	14.91 ± 0.67 fg	31.31 ± 2.56 a	27.92 ± 0.29 b	11.12 ± 0.29 h	15.28 ± 0.14 fg	16.77 ± 0.32 ef	20.80 ± 0.89 bc	18.48 ± 0.71 de
EC	11.73 ± 0.70 b	7.60 ± 0.14 d	9.04 ± 0.28 c	5.12 ± 0.14 e	11.65 ± 0.58 b	13.95 ± 0.40 a	9.73 ± 0.68 c	9.15 ± 0.74 c	8.59 ± 0.30 cd	7.55 ± 0.20 d	5.09 ± 0.17 e	11.44 ± 0.21 b	12.43 ± 0.39 b	11.41 ± 0.28 b
EGC	72.83 ± 5.50 abcd	71.60 ± 1.42 bcd	50.99 ± 1.45 g	65.09 ± 2.37 def	76.43 ± 2.91 abc	81.44 ± 2.20 a	80.21 ± 3.28 ab	49.92 ± 3.92 g	42.48 ± 0.65 g	69.68 ± 3.09 cde	60.53 ± 2.29 f	64.80 ± 1.81 def	73.84 ± 3.98 abc	61.44 ± 1.89 ef
EGCG3″Me	4.81 ± 0.22 c	6.48 ± 0.36 b	6.89 ± 0.49 b	0.03 ± 0.00 f	12.05 ± 0.45 a	2.62 ± 0.15 e	4.49 ± 0.05 c	0.03 ± 0.00 f	0.05 ± 0.00 f	0.03 ± 0.00 f	6.89 ± 0.39 b	0.13 ± 0.01 f	4.77 ± 0.18 c	3.38 ± 0.18 d
GC	2.48 ± 0.14 d	4.80 ± 0.16 b	2.45 ± 0.05 d	3.84 ± 0.21 c	4.00 ± 0.16 c	3.81 ± 0.05 c	3.81 ± 0.33 c	2.75 ± 0.30 d	1.44 ± 0.00 e	3.81 ± 0.09 c	2.67 ± 0.05 d	2.69 ± 0.12 d	6.75 ± 0.47 a	1.55 ± 0.05 e
C	0.75 ± 0.05 de	0.69 ± 0.12 def	0.85 ± 0.05 cd	0.69 ± 0.05 def	0.96 ± 0.08 c	1.23 ± 0.05 b	0.96 ± 0.08 c	0.77 ± 0.05 cde	0.67 ± 0.05 def	0.61 ± 0.05 ef	0.37 ± 0.05 g	0.83 ± 0.05 cd	1.76 ± 0.08 a	0.53 ± 0.05 fg
Rutin	0.68 ± 0.02 bcd	0.38 ± 0.01 de	0.89 ± 0.12 b	0.40 ± 0.04 de	0.44 ± 0.02 de	0.70 ± 0.07 bcd	0.60 ± 0.04 bcd	0.50 ± 0.07 cde	0.18 ± 0.02 e	0.42 ± 0.01 de	5.40 ± 0.39 a	0.83 ± 0.11 bc	0.85 ± 0.08 bc	0.23 ± 0.02 e
Caffeine	26.00 ± 2.28 b	18.35 ± 0.40 de	21.81 ± 0.44 cd	29.79 ± 1.40 a	23.97 ± 1.09 bc	25.41 ± 1.11 b	20.93 ± 1.38 cd	19.28 ± 1.42 de	22.93 ± 0.62 bc	14.27 ± 1.02 f	13.81 ± 0.58 f	14.16 ± 0.76 f	18.85 ± 1.72 de	16.51 ± 0.36 ef
**Amino acids**	**4.60 ± 0.32 g**	**6.01 ± 0.36 ef**	**21.07 ± 0.80 a**	**15.83 ± 0.72 c**	**6.68 ± 0.31 ef**	**6.84 ± 0.06 ef**	**5.83 ± 0.22 f**	**3.11 ± 0.15 h**	**18.24 ± 0.41 b**	**4.29 ± 0.39 g**	**4.33 ± 0.17 g**	**5.77 ± 0.35 f**	**8.05 ± 0.23 d**	**7.09 ± 0.19 de**
l-Theanine	2.61 ± 0.30 ef	3.31 ± 0.33 de	14.32 ± 0.68 a	12.08 ± 0.77 b	4.35 ± 0.36 cd	3.97 ± 0.05 cd	3.63 ± 0.28 de	0.80 ± 0.08 g	11.63 ± 0.62 b	1.81 ± 0.23 fg	2.08 ± 0.08 f	2.59 ± 0.26 ef	4.91 ± 0.30 c	4.13 ± 0.18 cd
Glu	1.15 ± 0.05 g	1.73 ± 0.10 cde	2.53 ± 0.10 b	2.33 ± 0.11 b	1.38 ± 0.07 fg	1.82 ± 0.07 cd	1.41 ± 0.09 fg	1.22 ± 0.05 g	3.05 ± 0.16 a	1.57 ± 0.08 def	1.45 ± 0.08 efg	1.76 ± 0.15 cd	1.68 ± 0.05 cdef	1.93 ± 0.17 c
Asp	0.22 ± 0.03 e	0.43 ± 0.01 d	1.13 ± 0.05 b	0.56 ± 0.02 c	0.27 ± 0.01 e	0.42 ± 0.03 d	0.22 ± 0.02 e	0.43 ± 0.02 d	1.37 ± 0.06 a	0.41 ± 0.05 d	0.23 ± 0.02 e	0.55 ± 0.01 c	0.55 ± 0.03 c	0.31 ± 0.04 e
Ser	0.22 ± 0.01 h	0.23 ± 0.03 gh	0.39 ± 0.02 c	0.35 ± 0.04 cde	0.29 ± 0.02 efg	0.22 ± 0.02 gh	0.24 ± 0.01 gh	0.28 ± 0.01 efgh	0.58 ± 0.01 a	0.24 ± 0.04 gh	0.24 ± 0.02 fgh	0.37 ± 0.02 cd	0.48 ± 0.03 b	0.31 ± 0.02 def
Trp	0.06 ± 0.00 a	0.02 ± 0.00 def	0.05 ± 0.00 b	0.05 ± 0.00 b	0.03 ± 0.00 c	0.05 ± 0.00 b	0.03 ± 0.00 cde	0.02 ± 0.00 fgh	0.03 ± 0.00 cd	0.01 ± 0.00 h	0.01 ± 0.00 gh	0.01 ± 0.00 gh	0.03 ± 0.00 cd	0.02 ± 0.00 efg
Phe	0.05 ± 0.00 bc	0.04 ± 0.00 bcde	0.05 ± 0.00 bc	0.04 ± 0.00 def	0.03 ± 0.00 efg	0.05 ± 0.00 bc	0.04 ± 0.00 bcde	0.05 ± 0.00 b	0.12 ± 0.01 a	0.04 ± 0.00 cdef	0.03 ± 0.00 g	0.03 ± 0.00 fg	0.03 ± 0.00 g	0.04 ± 0.00 bcd
Gln	0.05 ± 0.00 g	0.07 ± 0.01 fg	0.18 ± 0.01 c	0.16 ± 0.02 cd	0.10 ± 0.00 ef	0.12 ± 0.01 de	0.07 ± 0.00 fg	0.10 ± 0.01 ef	1.19 ± 0.05 a	0.07 ± 0.01 fg	0.10 ± 0.00 ef	0.27 ± 0.01 b	0.23 ± 0.01 b	0.14 ± 0.00 de
Leu	0.04 ± 0.00 b	0.02 ± 0.00 ef	0.07 ± 0.00 a	0.04 ± 0.00 bc	0.03 ± 0.00 cd	0.02 ± 0.00 efg	0.03 ± 0.00 ef	0.03 ± 0.00 ef	0.03 ± 0.00 de	0.02 ± 0.00 i	0.03 ± 0.00 de	0.02 ± 0.00 hi	0.02 ± 0.00 ghi	0.02 ± 0.00 fgh
Lys	0.04 ± 0.00 b	0.02 ± 0.00 defgh	0.15 ± 0.01 a	0.02 ± 0.00 cd	0.02 ± 0.00 de	0.02 ± 0.00 defgh	0.02 ± 0.00 defg	0.02 ± 0.00 def	0.03 ± 0.00 bc	0.01 ± 0.00 h	0.01 ± 0.00 efgh	0.01 ± 0.00 fgh	0.01 ± 0.00 gh	0.02 ± 0.00 de
Tyr	0.03 ± 0.00 b	0.02 ± 0.00 bc	0.02 ± 0.00 cde	0.01 ± 0.00 hi	0.02 ± 0.00 bcd	0.02 ± 0.00 def	0.02 ± 0.00 bc	0.02 ± 0.00 bcd	0.02 ± 0.00 efg	0.02 ± 0.00 fg	0.03 ± 0.00 a	0.02 ± 0.00 gh	0.01 ± 0.00 i	0.02 ± 0.00 fg
Pro	0.02 ± 0.00 cde	0.02 ± 0.00 f	0.03 ± 0.00 bc	0.03 ± 0.00 b	0.02 ± 0.00 ef	0.03 ± 0.00 bc	0.02 ± 0.00 def	0.03 ± 0.00 bcd	0.03 ± 0.00 a	0.02 ± 0.00 g	0.03 ± 0.00 bc	0.03 ± 0.00 bcd	0.03 ± 0.00 bc	0.02 ± 0.00 def
Thr	0.02 ± 0.00 bcde	0.02 ± 0.00 cde	0.03 ± 0.00 ab	0.03 ± 0.01 abcd	0.03 ± 0.01 abcd	0.02 ± 0.00 e	0.02 ± 0.00 bcde	0.02 ± 0.00 de	0.04 ± 0.01 a	0.03 ± 0.00 abcd	0.02 ± 0.00 cde	0.03 ± 0.00 abc	0.03 ± 0.00 bcde	0.03 ± 0.00 abcd
Val	0.02 ± 0.00 ab	0.01 ± 0.00 fg	0.02 ± 0.00 a	0.02 ± 0.00 cde	0.01 ± 0.00 defg	0.02 ± 0.00 bcd	0.02 ± 0.00 bc	0.02 ± 0.00 ab	0.01 ± 0.00 g	0.01 ± 0.00 efg	0.02 ± 0.00 bcd	0.01 ± 0.00 cdef	0.01 ± 0.00 efg	0.01 ± 0.00 cdef
GABA	0.02 ± 0.00 fg	0.02 ± 0.00 ef	0.11 ± 0.00 a	0.05 ± 0.00 b	0.03 ± 0.00 cd	0.02 ± 0.00 e	0.03 ± 0.00 d	0.01 ± 0.00 fgh	0.01 ± 0.00 i	0.01 ± 0.00 hi	0.01 ± 0.00 gh	0.03 ± 0.00 c	0.01 ± 0.00 hi	0.03 ± 0.00 d
His	0.02 ± 0.00 bc	0.01 ± 0.00 d	0.06 ± 0.01 a	0.01 ± 0.00 cd	0.01 ± 0.00 cd	0.01 ± 0.00 cd	0.01 ± 0.00 d	0.01 ± 0.00 d	0.02 ± 0.00 b	0.01 ± 0.00 d	0.01 ± 0.00 d	0.01 ± 0.00 d	ND	0.01 ± 0.00 d
Ile	0.01 ± 0.00 de	0.01 ± 0.00 efg	0.03 ± 0.00 a	0.01 ± 0.00 fg	0.02 ± 0.00 cd	0.01 ± 0.00 fg	0.01 ± 0.00 fg	0.01 ± 0.00 fg	0.01 ± 0.00 def	0.01 ± 0.00 gh	0.02 ± 0.00 bc	0.01 ± 0.00 gh	0.01 ± 0.00 h	0.02 ± 0.00 b
Arg	0.01 ± 0.00 b	0.02 ± 0.00 b	1.73 ± 0.06 a	0.03 ± 0.01 b	0.01 ± 0.00 b	0.01 ± 0.00 b	0.01 ± 0.00 b	0.02 ± 0.00 b	0.03 ± 0.00 b	ND	0.01 ± 0.00 b	0.01 ± 0.00 b	ND	0.02 ± 0.00 b
Asn	0.01 ± 0.01 b	0.01 ± 0.00 b	0.16 ± 0.02 a	0.02 ± 0.00 b	0.01 ± 0.01 b	0.01 ± 0.01 b	0.01 ± 0.01 b	0.01 ± 0.00 b	0.03 ± 0.01 b	0.01 ± 0.00 b	0.01 ± 0.00 b	0.01 ± 0.01 b	0.01 ± 0.00 b	0.01 ± 0.00 b

Results are expressed as mean ± standard deviation (*n* = 3). Means with different letters in row are significantly different according to Tukey’s HSD test (*p* < 0.05). ND = non-detectable.

## Supplementary Materials

The Supplementary Materials are available online, Figure S1: Distribution of metabolites in the methanol extracts of fresh leaves of 14 Wuyi Rock tea cultivars, Table S1: Filtered and normalized PCA data matrix generated from UPLC-QTOF MS in ESI^−^.
