# Supplementary material for: Metabolite Profiling of 14 Wuyi Rock Tea Cultivars Using UPLC-QTOF MS and UPLC-QqQ MS Combined with Chemometrics
Source: Molecules. 2018 Jan 24;23(2):104. doi: 10.3390/molecules23020104 (PMC6017971; doi:10.3390/molecules23020104)
Supplement: Supplementary file 1 [file molecules-23-00104-s001.zip › Figure S1.docx]

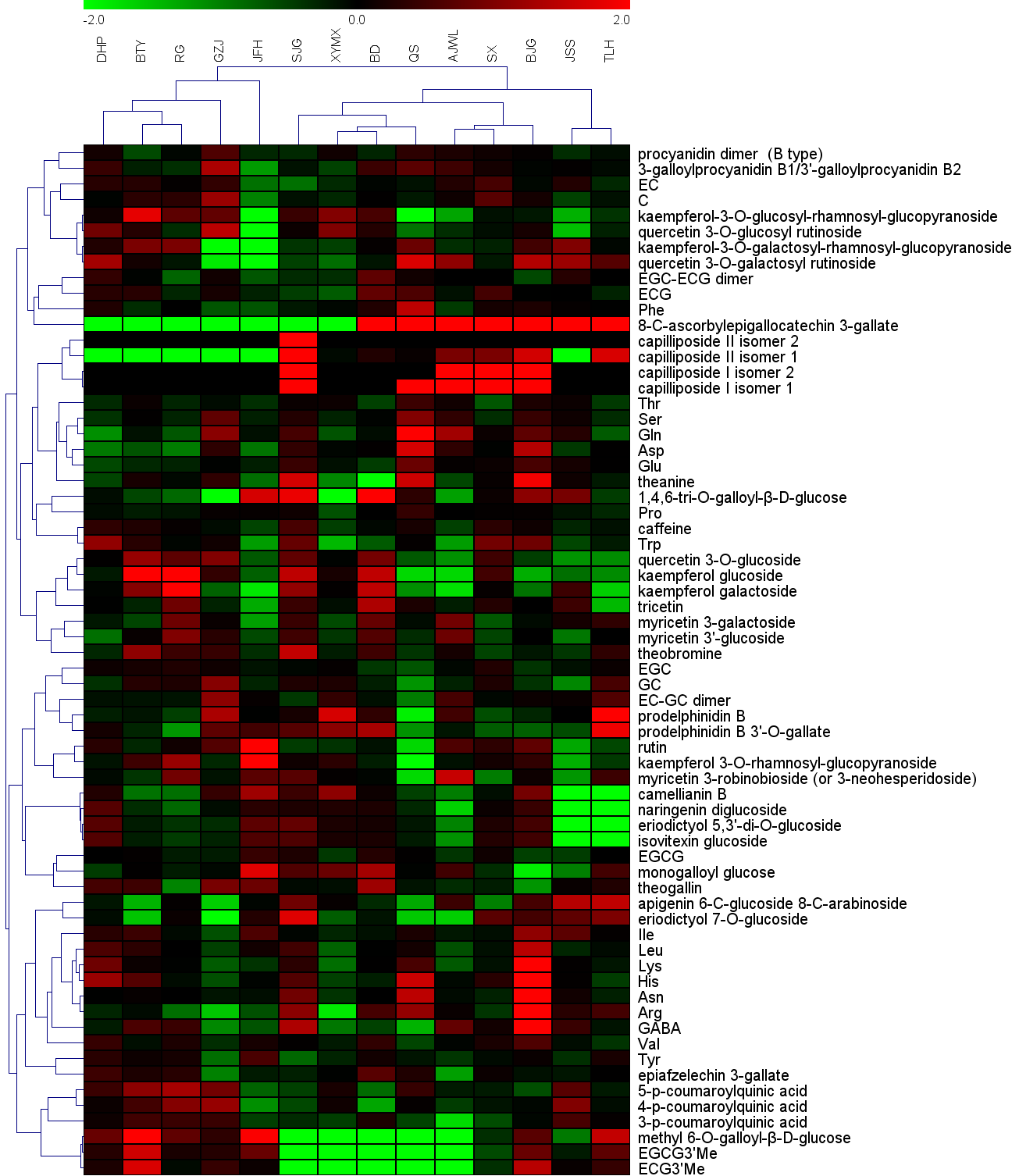


**Figure S1**. Distribution of metabolites in the methanol extracts of fresh leaves of 14 Wuyi Rock tea cultivars. The analysis is based on the normalized average signal abundance from three biological replicates for each cultivar. Heat map with hierarchical clustering (Pearson’s correlation, average linkage) was generated with MultiExperiment Viewer software (version 4.9.0) to visualize accumulation patterns of annotated major metabolites between sample types.
